# Radiation Damage by Heavy Ions in Silicon and Silicon Carbide Detectors

**DOI:** 10.3390/s23146522

**Published:** 2023-07-19

**Authors:** Carmen Altana, Lucia Calcagno, Caterina Ciampi, Francesco La Via, Gaetano Lanzalone, Annamaria Muoio, Gabriele Pasquali, Domenico Pellegrino, Sebastiana Puglia, Giuseppe Rapisarda, Salvatore Tudisco

**Affiliations:** 1 Laboratori Nazionali del Sud (LNS), Istituto Nazionale di Fisica Nucleare (INFN), 95123 Catania, Italy; altana@lns.infn.it (C.A.); lanzalone@lns.infn.it (G.L.); grapisarda@lns.infn.it (G.R.); 2 Istituto Nazionale di Fisica Nucleare (INFN)—Sezione di Catania, 95123 Catania, Italy; lucia.calcagno@ct.infn.it (L.C.); sebastiana.puglia@dfa.unict.it (S.P.); 3 Physics and Astronomy Department, Catania University, 95123 Catania, Italy; domenico.pellegrino@dfa.unict.it; 4 Physics and Astronomy Department, Florence University, 50019 Florence, Italy; ciampi@fi.infn.it (C.C.); pasquali@fi.infn.it (G.P.); 5 Istituto Nazionale di Fisica Nucleare (INFN)—Sezione di Firenze, 50019 Florence, Italy; 6 Institute for Microelectronics and Microsystems (IMM), National Research Council (CNR), 95121 Catania, Italy; francesco.lavia@imm.cnr.it (F.L.V.); annamaria.muoio@imm.cnr.it (A.M.); 7 Department of Engineering and Architecture, KORE University, Cittadella Universitaria, 94100 Enna, Italy

**Keywords:** radiation hardness, silicon, silicon carbide, collection efficiency, energy resolution, defects, DLTS

## Abstract

While silicon has been a steadfast semiconductor material for the past 50 years, it is now facing competition from other materials, especially for detector design. In that respect, due to its high resistance to radiation damage, silicon carbide is one of the most promising materials. In this work, we discuss the radiation damage studies of a new, large area, p-n junction silicon carbide device developed by the SiCILIA collaboration. We have studied the general performances of several devices, as a function of fluence, irradiated in different experimental conditions with different beams. A standard p-n junction silicon detector was also irradiated for comparison. The new detectors manifest excellent performance in terms of stability of the main parameters, linearity, defect distribution, charge collection efficiency, energy resolution, leakage current, etc. Experimental results evidence a radiation resistance of SiC devices more than two order of magnitude higher than Si devices. The new construction technology applied to silicon carbide material has made it possible to create very robust devices with excellent performance. These devices will soon be available for all those scientific projects where a high resistance to radiation damage is required.

## 1. Introduction

Silicon Carbide (SiC) represents a new challenge for detector manufacturing [1]. The frontier activities of nuclear and subnuclear physics require devices with excellent performance in terms of stability and ability to operate at high fluxes of incident particles, e.g., for measuring cross-sections of very rare phenomena [2]. SiC is extremely appealing for its expected features in terms of radiation hardness; it is a wide band gap semiconductor and is presently the most intensively studied because it is a good alternative to silicon as radiation hard device [3,4,5,6]. The irradiation is known to cause an appreciable deterioration of the detector performance [7,8]. Generally, the main evidence of damage is a fluence proportional increase of the leakage current. Loss of energy resolution and of charge collection efficiency are also signs of increasing damage. All these effects have been measured so far in an impressive number of systematic studies on Silicon [9].

SiC p-n junctions should be potentially very robust devices from a radiation hardness point of view. Innovative detection systems construction for the new nuclear physics experiments at high beam luminosity, requires the development of large area devices able to survive the exposure of highest radiation levels. The requests of new and ambitious projects of the Italian National Institute of Nuclear Physics—INFN (NUMEN [10], NuReLP [11,12,13,14]), led a cooperation between INFN and IMM-CNR, started in 2016, in order to develop a R&D activity on SiC technology. The SiCILIA project (Silicon Carbide detectors for Intense Luminosity Investigations and Applications) [15] has been totally funded by INFN. Results on technological issues and particle detection applications have been recently published [16,17,18].

In this work, we study the effects of damage in SiC from the point of view of both the electrical characteristics and the spectroscopic behavior as a nuclear particle detector. Radiation damage studies were conducted at the Laboratori Nazionali del Sud (INFN) in Catania, Italy, by using the ion beams delivered by the 15 MV Tandem accelerator in two different measurement sets. The Devices Under Test (DUTs) were mounted in the CT-2000 chamber, which is placed at the end of the 60-degree beam line. The chamber, with a diameter of 2 m, is equipped with 2 independently rotating arms to support detectors and with different collimation systems, allowing to measure precise angular distributions.

In a first experimental campaign, the DUTs were irradiated with ^16^O ions at 12.5 MeV, together with a standard silicon detector, for comparison. The DUTs were collimated so that just a small portion of the device was irradiated. In this way we have removed the effect of the different structure of the detectors, and only the effect of the different materials can be observed in this kind of experiment. To investigate the variation of the electrical characteristics properties as a function of the irradiation fluence, a second set of measurements was performed, with only the SiC detectors, in which we irradiated the whole device to verify its behavior under common operating conditions. Furthermore, due to the high radiation hardness of the SiC detectors observed in the previous experiment, it has been decided to use a heavier ion and the irradiation has been performed with ^27^Al.

The paper is organized as follows. After a brief recollection of the relevant SiC characteristics (Section 2), the devices studied in this work are described in Section 3. Results of the first measurement set, employing ^16^O for damaging the detectors, will be discussed in Section 4. Section 5 presents the results of the second set, where ^27^Al has been used. While Section 4 and Section 5 deal mostly with effects of radiation damage on the detection characteristics of SiC, Section 6 presents a more detailed structural study of the behavior of the DUTs irradiated with ^27^Al: the produced defects are characterized using the Deep Level Transient Spectroscopy (DLTS) technique and changes in the current-voltage characteristics are also reported. The last section (Section 7) is devoted to summarizing the work and drawing some conclusions.

## 2. Silicon Carbide Properties

SiC exists in more than 200 different polytypes, in which it can assume a wide variety of crystallographic structures (polytypism) depending on the stacking sequence of the atoms; the most common structures used for microelectronic applications are 3C [19,20], 4H and 6H [21]. 4H-SiC is largely used in radiation hard applications [22,23,24,25].

Though the substrate material has recently improved both in size and purity, the present quality and uniformity of the epitaxial layers does not allow the processing of devices directly on it. Therefore, first of all, it is necessary to grow an epitaxial layer on a substrate that acts as the active region of the SiC detector. A seed substrate with very low defect concentration and the reduction of the density of defects present in the substrates by means of an epitaxial process allow a high-quality material to be obtained. The growth rate condition also has a significant influence on the defect formation or annihilation. Consequently, all these parameters are essential to obtain detectors with large area and thick active regions. Recently, due to the demands on the material quality and throughput increases, the chemical vapor deposition (CVD) as epitaxial growth technique was chosen. In the last decades, important progress on the epitaxial and hetero-epitaxial processes of silicon carbide has been made: introduction of chloride precursors [26,27], epitaxial growth on large area substrate with low defect density, improvement of the surface morphology, understanding of the CVD reactions and epitaxial mechanisms by advanced simulations [28] point out the main results obtained in the homo-epitaxy process of 4H-SiC [29]. Such evolution is a fundamental step towards the detectors manufacturing technology where large detection surfaces and thick devices are required.

Bulk damage is considered the limiting factor for the use of semiconductor detectors in the intense radiation field [30]; hadrons or energetic leptons produce bulk damages primarily due to displacing a Primary Knock-on Atom (PKA) out of its lattice site. The process energy threshold, for several kinds of semiconductor materials, is reported in Table 1; 4H-SiC epilayers exhibit an intermediate energy value in between Silicon and Diamond.

The pair of a Silicon (or Carbon) interstitial and a vacancy (denominated Frenkel pair) is a single displacement that can migrate through the lattice and may form point defects or agglomerate to form extended defects (particularly dislocations). The energy of a recoil PKA or residual atoms resulting from a nuclear reaction can be much higher. The energy loss along the paths of the recoils is due to two competing contributions: the collision with atomic electrons (collisional losses) and other displacements. Non-collisional interactions are predominant at the end of heavy recoil range and a dense agglomerate of defects (clusters) is formed. The bulk damage effects in semiconductor detectors are due to both the point defects along the incident particle paths and clusters at the end of the particles range. The detector performance is affected by point defects and clusters of point defects, depending on their concentration, energy level and the respective electron and hole capture cross-section. Defects that have deep energy levels in the middle of the forbidden gap could act as recombination/generation centers and to lead an increase of the reverse detector current. The effective doping concentration and the operating voltage needed to fully deplete the detector thickness change because of the generation of charged centers and the removal of dopants by formation of complex defects. These defects could also affect the carge collection efficiency by acting as trapping centers. In that respect the larger energy threshold to produce a PKA makes SiC a valid alternative to silicon.

Another advantage of SiC devices with respect to Silicon is the much smaller leakage current due essentially to the larger band gap [31] (the reverse current density of SiC p-n junction is expected to be three order of magnitude smaller than for Si).

## 3. New Large Area p-n Junctions SiC Devices

**Figure 1:**
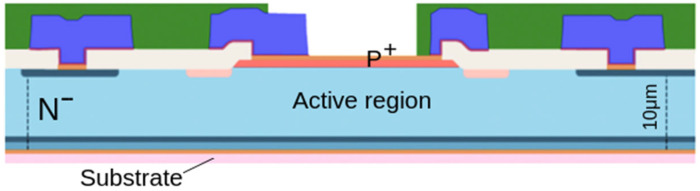
Schematic representation of 10 μm thick detector.

The detectors studied in this work are new large area devices developed, during the SiCILIA project, by a collaboration between INFN, IMM and ST-Microelectronic. They are produced starting from 10–100 μm n^−^ epi-layers grown on 350 μm substrates. A cross section of the device is shown in Figure 1. The first step of the processing is the growth of a double epitaxial layer, necessary for the p^+^/n junction implementation. A p^+^ layer 0.3–0.5 μm thick with an aluminum doping concentration of the order of 10^18^–10^19^/cm^3^ is grown over the n^−^ epi-layer with a nitrogen doping concentration in the range 5–8 10^13^/cm^3^ (Figure 1). After these phases, a first photolithography follows for the definition of the detector area by an ICP plasma etching and a second and a third lithography for the construction of the edge structure [15]. This edge structure is needed to reduce the electric field at the device borders, and in particular, it is implemented by using a junction termination extension (JTE) by Al^+^ implantation. Then, a third lithography is performed to realize by P^+^ ion implantation a n^+^ region on the edge of the device that acts as a field stop. After these two implants, a high temperature process at 1650 °C activates the dopants. The processing continues with the deposition of an isolation oxide and the opening of the contacts with a further photolithographic process. Then, the front metallization (Ni) is deposited and subsequently annealed to form a good ohmic contact on the p^+^ and the n^+^ region. The unreacted Ni on the oxide is then removed by a selective etch, and only on the periphery of the detector a thicker layer of Ti and Al is deposited for the bonding of the detector [15]. After the lithography to define the metal layer, a thick polyamide layer is deposited for the passivation, and a further lithography is performed for the opening of the detector active area. Finally, the substrate is reduced from the backside by a mechanical process until a final thickness of the epitaxial layer plus the substrate of 110 μm, and the ohmic contact is formed by a titanium/nickel/gold deposited on the back of the wafer.

The active thickness of the devices tested in this work is about 10 μm. Then the depletion bias is about 4 V. The detector area is 5 × 5 mm^2^.

After the realization of the entire process, the detectors have been tested, cut off and mounted on a board for testing with ions.

## 4. Charge Collection Efficiency in SiC and Si When Damaged with ^16^O

**Figure 2:**
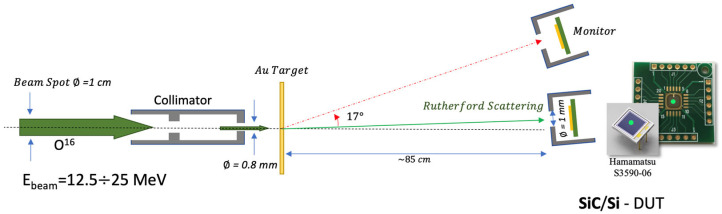
Irradiation and CCE measurement setup used for the ^16^O beam. A SiC detector placed at 17° with respect to the beam direction is used as beam monitor. The DUT is placed at a smaller angle (about 1°) to be irradiated with the ^16^O ions elastically scattered by the Au target.

The effects of the irradiation on the performances of SiC when used as a spectroscopic detector are of particular interest for nuclear physics applications. In particular, in this section, we study the charge collection efficiency and the resolution degradation of the DUTs as a function of the ion fluence in the first measurement set performed by SiCILIA. In this set, the DUTs were placed in the CT-2000 vacuum chamber at about 85 cm from the target. A schematic view of the setup is shown in Figure 2. The ^197^Au target is about 100 µg/cm^2^ thick, placed normal to the beam direction. The beam energy is 12.5 MeV. The ^16^O beam is collimated to less than 1 mm before impinging on a ^197^Au target. The DUTs are placed at angles ranging from 0.5° to 10° with respect to the beam and collimated with a 1 mm diameter collimator to protect the edge structures from radiation damage effects. The ions enter the detectors from the junction (p^+^) side. Each DUT is irradiated at a different fluence, ranging from 8.8 10^7^ pp/cm^2^ to 4.2 10^11^ pp/cm^2^, by placing the DUTs at different angles depending on the desired fluence. Different DUTs are used to evaluate the reproducibility of the results. In the same geometrical conditions, a silicon detector is irradiated at a larger energy (25 MeV) with the aim to compare the damage effects in the two different materials. In the first part of the study, the Tandem accelerator was set to deliver a high energy beam useful for the calibration of Si device; in such conditions, the radiation damage on Si detector was investigated. In the second part of the study, the energy of the ^16^O beam was reduced at 12.5 MeV to make the beam ions stop within the 10 μm of SiC and proceed with its investigation.

The SiC devices have been presented in Section 3. The silicon detector used for comparison is an Hamamatsu S3590-06 p-i-n chip, 300 µm thick. Since the beam is stopped both in the SiC and in the Si detector, the larger thickness of the latter does not subtract from our conclusions in terms of radiation damage.

Standard spectroscopic electronics is used to process the signals: charge preamplifiers of 45 mV/MeV gain from the ASCOM company [32], a spectroscopy amplifier (Mesytec Corporation mod. MSCF-16-F, Putzbrunn, Germany), set to 2 µs shaping time and a Silena 4418/V peak sensing ADC. The much larger capacitance of the SiC detector is associated with a larger electronic noise contribution to its energy resolution, with respect to the p-i-n silicon. However, electronic noise is not the dominant contribution to the energy resolution in our case.

The beam current is evaluated by using a pristine SiC detector used as a monitor and placed at 17° with respect to the beam direction (the beam current can be easily calculated from the detector active area and its counts per second, taking into account the Rutherford scattering cross section). From the beam current and the irradiation time, we evaluated the fluence for each DUT, again considering the Rutherford cross section at the corresponding polar angle. The fluence on the monitor detector is 10^3^–10^5^ times smaller than that on the DUTs, depending on the angle, so that the monitor does not experience any appreciable damage.

**Figure 3:**
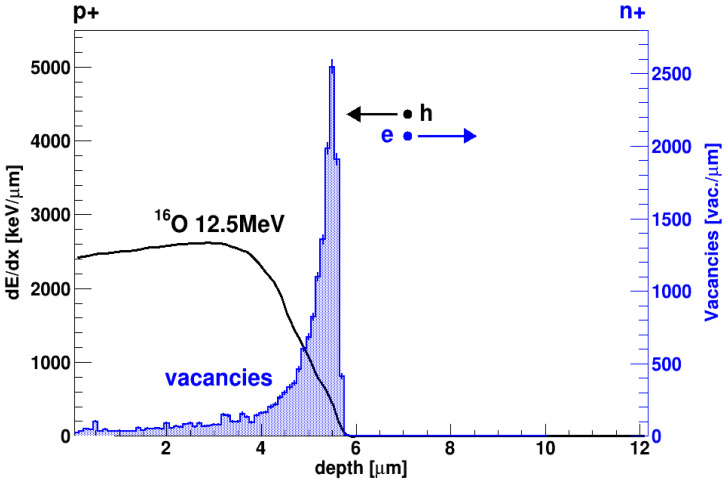
Black solid line: Bragg curve (stopping power vs. penetration depth) for ^16^O at 12.5 MeV in SiC. Blue-filled histogram: number of vacancies created for unit length. Both histograms have been obtained using SRIM 2013 [33].

It is important to notice that, in this experiment, the Charge Collection Efficiency (CCE) is studied with the same ions producing the damage. Figure 3 shows the distribution of the defects (vacancies) produced in SiC by a 12.5 MeV ^16^O ion, according to a SRIM [33] simulation. Since the range of ^16^O ions in SiC is 5.6 µm, it is clear from the picture that most damage is produced at the very end of the ion range. The specific energy loss due to collisions with electrons is also reported (Bragg curve): the linear density of the electron-hole pairs produced by the ion is proportional to this value. After the ionization track is produced, electrons drift to the right, towards the n+ electrode; conversely, holes drift to the left, towards the p+ electrode. It is thus clear that electrons have to pass through the most damaged region in order to reach their collection electrode, after which they go through a relatively undamaged region. Holes, on the other end, drift all along the damaged region, though only part of them go through the most damaged zone.

**Figure 4:**
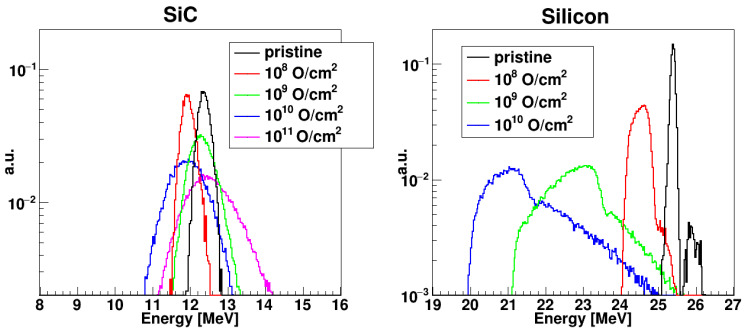
Energy spectra of ^16^O beams for SiC at 12.5 MeV (**left** panel) and Si at 25 MeV (**right** panel) for increasing values of ion fluence. For each fluence, the spectrum corresponds to the maximum CCE obtained. For each graph, counts are normalized to the total integral of the spectrum.

All detectors used for the investigation (Si and SiC) were preliminarily calibrated by using the ^16^O beam at different energies. Energy spectra of ^16^O beams for pristine and irradiated detectors are shown in Figure 4 for different values of the ion fluence. Spectra are taken at the bias voltage corresponding to the maximum Charge Collection Efficiency (CCE) obtained. The left panel refers to a SiC detector, the right one to the Si detector. Since the impinging radiation is monoenergetic, the spectra are characterized by a single peak. For the SiC detector, the peak centroids are close to each other, since a CCE of about 100% is reached at all the fluences except 10^12^ cm^−2^. However, the peaks get larger for increasing fluence, showing increasing fluctuations in the charge collection process. As a matter of fact, in an actual nuclear physics experiment, a constant CCE is of great advantage, since the same energy calibration could be applied to all the acquired data. It is evident, from Figure 4, that a constant CCE could be obtained for SiC, by suitably adapting the bias voltage, and not for silicon.

For silicon, the peak moves to a lower abscissa (i.e., we observe a reduction of the CCE) for increasing fluence, and already at 10^9^ ions/cm^2^, it develops a tail on the right side and a shoulder on the left side, becoming asymmetric. Beyond 10^10^ ions/cm^2^, the detector stops working at all. Furthermore, the leakage current of the silicon detector increases considerably (from 0.2 μA up to 2 μA), even after the lowest dose value.

**Figure 5:**
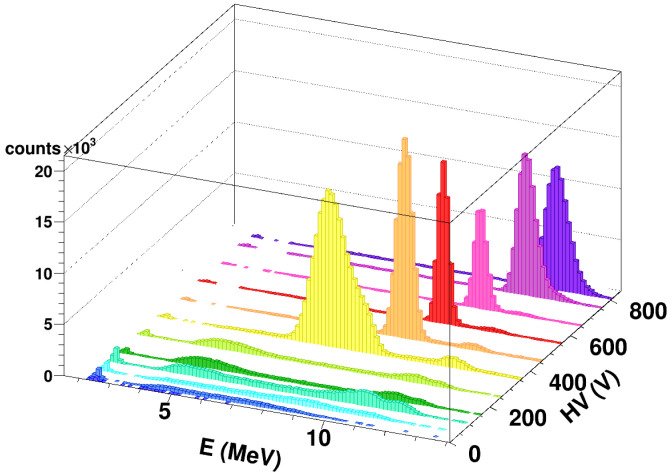
Calibrated energy spectra obtained at different bias voltage values for a SiC detector irradiated with ^16^O beams at 12.5 MeV (10^9^ cm^−2^ fluence). Different colors help the readability, expecially when histograms partially overlap.

By studying the CCE of the SiC detector as a function of the applied voltage, one can notice that, starting at 10^9^ cm^−2^ fluence, a voltage of at least 300 V is needed to get a peak in the amplitude distribution. This behavior can be easily seen in Figure 5, which shows the obtained spectra for the different applied voltages at a 10^9^ cm^−2^ fluence, and it is not due to a slowed down collection time, which could produce ballistic deficit in the amplitude measurement: the rise-time of the preamplifier signal is much shorter than the shaping time for all applied voltages. Though the reason for this effect is still under study, the large reduction of CCE at low reverse bias could be due to the setting of the experiment. In fact, part of the detector has been damaged at high fluence with oxygen ions, and just after a few minutes, the CCE has been measured with the same ions. With this procedure, even the transient defects that have a very low activation energy as reported in several previous papers [34,35,36], can affect the CCE. During the CCE measurements, the annealing at room temperature produces a considerable reduction of the low activation energy traps, and then, a considerable increase of the CCE can be observed. A preliminary characterization with alpha sources has shown that, already a few days after irradiation, the CCE had substantially improved. Further studies are currently planned to quantify the effect.

**Figure 6:**
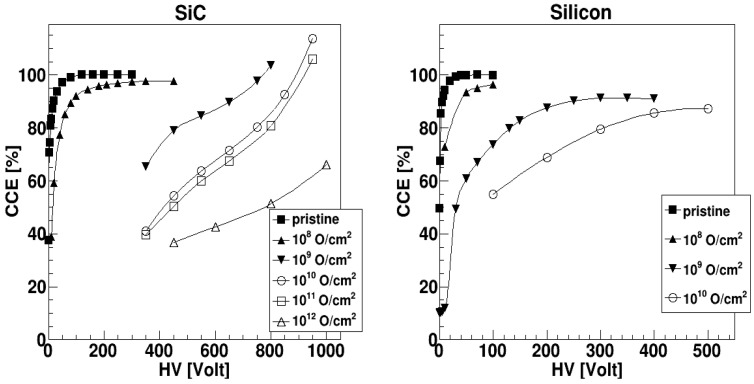
CCE values as a function of the bias voltage for SiC (**left** panel) and silicon (**right** panel) detectors at different fluences. For each detector, all points are normalized to the maximum CCE obtained in pristine conditions.

Figure 6 shows the obtained CCE values as a function of the applied voltage. The CCE has been calculated only for those voltage values for which a proper peak is present. The most striking difference between the SiC and the silicon data is the behavior at the largest fluence values. The silicon detector always shows a tendency to saturate with increasing voltage. For increasing fluence, we can observe both a reduction of the CCE and an increase of the bias to obtain the saturation value: already at 10^8^ cm^−2^ the saturation value stays well below 100%.

**Figure 7:**
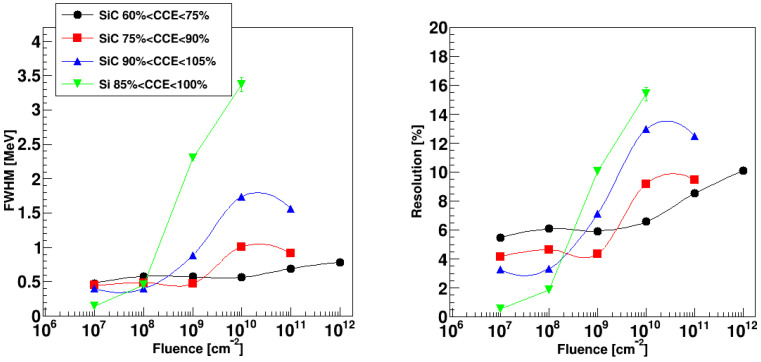
Full Width at Half Maximum (**left**) and resolution (**right**) for the SiC and Si detectors as a function of the fluence. For the SiC detectors, data corresponding to different intervals of CCE are presented (see legend). A Gaussian fit has been used for all cases except for those silicon detector peaks for which a large asymmetry suggested the use of the standard deviation of the whole distribution (multiplied times 2.35 for meaningful comparison with the FWHM).

The SiC detector, instead, shows an increasing CCE value, which even trespasses 100% at very high reverse bias (V > 800 V). This could be due to damage in the SiC material that produces a substantial change in the local doping concentration and, consequently, a peak of the electric field close to the electrodes and an impact ionization of the carriers in those regions. The impact ionization multiplication then gives a larger than 100% CCE, as already recognized in silicon detectors [37]. As can be seen in Figure 4, larger fluctuations are obtained at the largest fluences: This could be also due to the additional multiplication associated to the impact multiplication process. As a matter of fact, for the same fluences, the width of the distribution drastically decreases at lower bias voltages (associated to lower CCE values). This is also illustrated in Figure 7, where the FWHM of the distribution (left panel) and the associated resolution (right panel) are reported as a function of the fluence.

The data for SiC are also shown for different CCE intervals, while data for silicon refer to the largest CCE value. Data for SiC extend to fluence values one or two orders of magnitude larger than for silicon since the SiC still produces a single peak at those fluences. It is also clear that the worsening of the performance, in terms of FWHM and resolution, is much steeper for silicon than for SiC, though the initial resolution of the silicon detector is better. The SiC points at CCE~100% show a much worse resolution than for lower CCE values. This could be due to the already mentioned impact ionization effect at large bias voltage. As a matter of fact, the SiC energy resolution stays below 10% even at a 10^12^ cm^−2^ fluence, provided that the bias voltage is not increased too much.

## 5. Charge Collection Efficiency and Resolution in SiC Damaged by ^26^Al

In the second measurement set, for irradiation with ^27^Al at 24.2 MeV, three DUTs were placed in the target position at 90° with respect to the beam direction. At this energy, ^27^Al has a range of 6.9 µm in SiC, and it is therefore stopped in the active region. The beam was purposely defocused to irradiate the whole surface as uniformly as possible. The DUTs, nominally identical, were irradiated at different fluences, ranging from 10^9^ to 10^11^ pp/cm^2^. Before and after irradiation, each device was tested in vacuum, in terms of its spectroscopic performance, using two alpha sources: a ^148^Gd source (E = 3.183 MeV) and an ^241^Am source (main peak E = 5.486 MeV). The energy spectra were acquired with a standard spectroscopic electronic chain composed by a charge preamplifier of 45 mV/MeV gain from the ASCOM company [32], a spectroscopy amplifier (ORTEC mod. 572), set to 2 µs shaping time, and a multi-channel analyzer. Differently from the experiment described in Section 4, in the present measurement, the ion used to damage the detectors is not the same as that employed to characterize them. Moreover, the CCE was evaluated many hours after irradiation.

**Figure 8:**
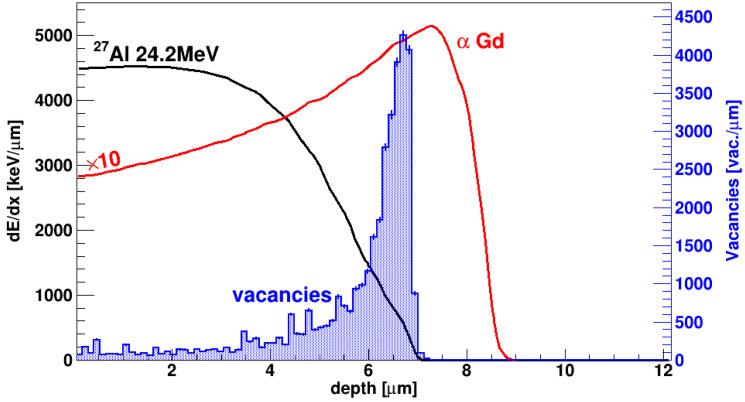
Black solid line: Bragg curve (stopping power vs. penetration depth) for ^27^Al at 24.2 MeV in SiC. Red solid line: Bragg curve for alpha particles at 3.183 MeV (Gd source) in SiC, multiplied by a factor of 10 to facilitate the comparison. Blue-filled histogram: number of vacancies created for unit length by ^27^Al at 24.2 MeV in SiC. All histograms have been obtained using SRIM 2013 [33].

Figure 8 shows the distribution of the vacancies produced by the ^27^Al ions, together with their ionization profile and the one of the alphas from the ^148^Gd source. It is apparent, from Figure 8, that for alpha particles the maximum density of the produced electron-hole pairs roughly corresponds to the maximum defect density.

The energy spectra of the ^148^Gd alpha source feature two peaks, one centered at 3.18 MeV and one centered at about 2.5 MeV. The former, relatively narrow, is associated with alpha particles impinging on the active area of the detector, where the only entrance window is due to the p+ electrode. The latter, much larger and slightly asymmetric, is due to alpha particles impinging closer to the borders of the active area, where a relatively thick passivation (7 µm of polyamide) is present: the alpha particles experience an energy loss of 0.6–0.8 MeV in the passivation layer before entering the active area. The data presented in this paper were obtained using only the “clean” peak not affected by the passivation.

The energy spectra have been calibrated for DUTs in pristine conditions, also using an ^241^Am alpha source. The alpha particles from the ^241^Am source, having a range of about 18 µm in SiC, are not stopped in the active region of the SiC detectors. Using both sources and both peaks (the “clean” peak and the one due to the passivation layer), we have developed a self-consistent calibration procedure, based on energy loss calculations. The procedure gives the calibration parameters for each spectrum and estimates the average dead layer thickness (about 2 µm SiC equivalent) and the active region thickness, the latter resulting slightly larger (12–13 µm) than the nominal value of 10 µm.

**Figure 9:**
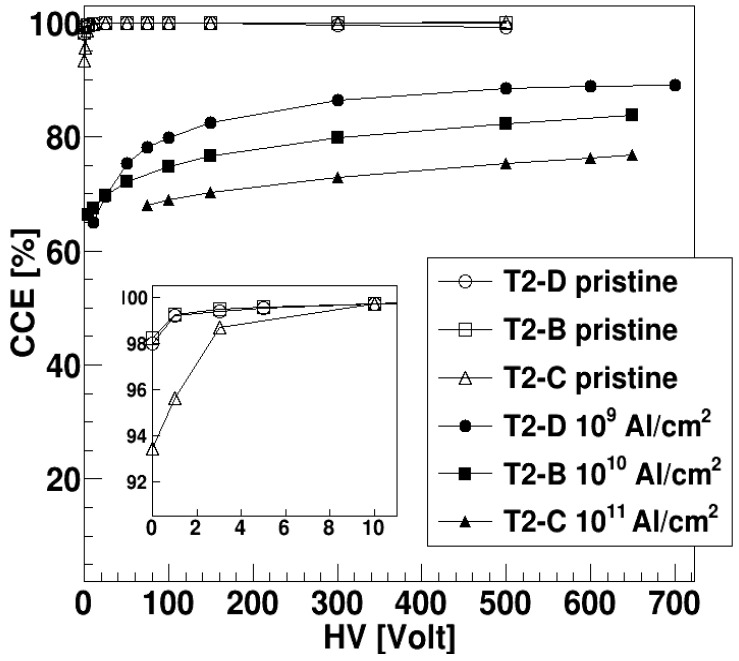
CCE for alphas from a ^148^Gd source as a function of the applied bias voltage. For each DUT, CCE values before and after irradiation are shown. The inset shows an expanded view of the top left data, for a better readability.

**Figure 10:**
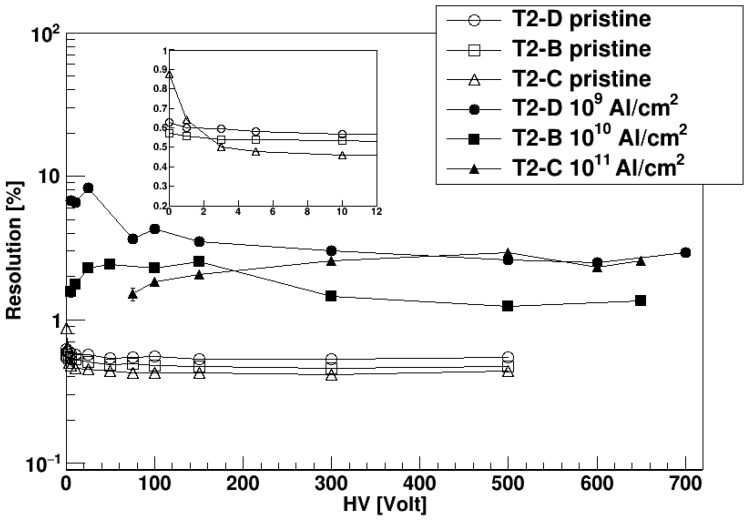
Energy resolution for alpha particles from a ^148^Gd source as a function of the applied bias voltage. For each DUT, values before and after irradiation are shown. The inset shows an expanded view of the low voltage part for a better readability of the pristine data.

An analysis of the CCE has been performed for both alpha sources, and data obtained with the two sources are compatible. Figure 9 shows the CCE obtained using the ^148^Gd source, as a function of the applied voltage. The CCE is calculated from the centroid of the “clean” alpha peak, in energy units, normalized to the maximum centroid obtained for the pristine detector. All the DUTs have practically the same behavior before irradiation with the CCE reaching 100% already at 10 V bias voltage (see the inset in Figure 10 where the values at low voltages for the pristine detectors are shown). The CCE decreases for increasing fluence. The bias voltage for which CCE saturates also tends to increase. This effect could be due to the decrease of the carrier lifetime produced by the damage in the silicon carbide epitaxial layer after the irradiation. For a given electric field, the carrier transit time is fixed, so that a decrease in the carrier lifetime produces a decrease of the CCE. By increasing the electric field, the transit time in the depletion layer is reduced so that a larger fraction of the charge can be collected. Even at the largest fluence (about 10^11^ cm^−2^), the CCE is reduced just by about 30%. Though a larger bias voltage is needed to saturate the CCE, after irradiation, the saturation value is already reached, within a few percent, at about 300 V. CCE is never larger than 100%, at variance with Figure 6, but in this case the measurements have been done after several days (so that some annealing could have occurred) and with a different ion and energy with respect to those used for irradiation.

The shape of the alpha peaks has been found symmetric and Gaussian also after irradiation. Both the Full Width at Half Maximum (FWHM) of the peak and its centroid get smaller after irradiation. Their ratio, i.e., the energy resolution, is the parameter of interest when one wants to measure an unknown energy spectra. In Figure 10 we report the resolution of the ^148^Gd alpha peak as a function of the bias voltage, for the three DUTs both before and after irradiation. Pristine detectors have very similar resolutions of ~0.5%. After irradiation, the resolution worsens by almost a factor of 10, reaching a few percent, a value in any case still acceptable in many of the foreseen applications. The fact that resolution values at 10^9^ cm^−2^ and 10^11^ cm^−2^ fluence are similar and larger than the value at 10^10^ cm^−2^ has come as a surprise, and it is still under study. The resolution is also only mildly dependent on the bias voltage, at least above ~50 V, and reaches a steady value already at 300 V.

## 6. DUTs Irradiated with ^27^Al: Electrical Characterization

**Figure 11:**
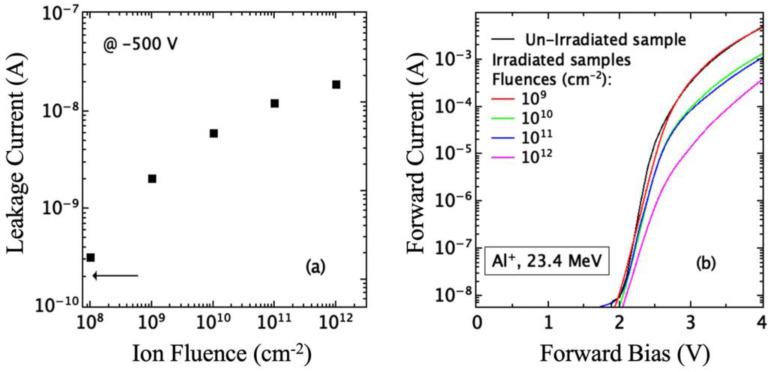
(**a**) leakage current measured at −500 V vs. ion fluence; (**b**) forward characteristics of different fluence irradiated devices. The arrow in (**a**) refers to the current measured in the unirradiated devices.

The forward and reverse I–V characteristics of the p-n junction have been measured before and after irradiation with 24.2 MeV Al ions at different fluences. The current–voltage measurement has been performed with the Keithley 6517 electrometer, Beaverton, OR, USA, which works as voltage source and as amperometer. The system has been interfaced to a PC through a GPIB interface. The reverse current measured at a bias of −500 V is reported in Figure 11a versus the ion fluence: In the unirradiated diode it is about 3 × 10^−10^ A (arrow in Figure 11a) and increases after irradiation reaching a value of 2 × 10^−8^ A at a fluence of 10^12^ ions/cm^2^. A similar effect was observed in Schottky diodes irradiated with 1.0 MeV Si [5], and it was associated with the defects produced by irradiation.

As shown in Figure 8, the energy loss and the vacancy concentration of 24.2 MeV Al ions, as calculated by SRIM simulator [33], are not uniform in the device active layer as the ion range (6.6 μm) is lower than the epilayer thickness (10 μm). The primary vacancy concentration at the end of the ion range is about a factor ten higher than in the first 5 μm, as shown in Figure 8. Then, in the first region of irradiated epilayer, only point defects (vacancy and interstitial) are produced instead around the ion range cluster of defects formed at least at high fluence [38].

We have measured the I–V forward characteristics of the p-n junction before and after irradiation, and in Figure 11b, the obtained results are reported. The unirradiated diode exhibits the typical behavior of a p-n junction, showing a turn-on voltage of about 1.8 V, an exponential increase of current with bias at low voltage and a linear increase at higher voltages (>2.5 V) due to presence of the series resistance [39]. After irradiation, these characteristics change resulting in a decrease of the current at fixed bias and a right shift of characteristic: the knee shifts at higher bias and the diode turn-on voltage changes from about 1.8 V to about 2.2 V after irradiation.

In the low voltage region, this characteristic can be described with the Shockley-Hall-Read (SHR) generation current and with the model reported by C.T. Sah et al. [40]. The current is related to the recombination in the space–charge region through deep levels near the middle of the bandgap. In that region, the current density can is given by:

J = J_0_ exp(qV/*n*K_B_T) (1)

where V is the applied bias, *n* the ideality factor, K_B_ the Boltzmann constant, q the electron charge and T the absolute temperature. The pre-exponential factor J_0_ in the relation (1) is obtained by the relation:

J_0_ = q n_i_w/τ_0_. (2)

where w is the width of the recombination region, n_i_ the concentration of intrinsic charge carriers and τ_0_ the carrier lifetime. However, the last parameter, τ_0_, is given by the well-known relation [31]:

(3) $$\tau_{0}=\frac{1}{N_{t}\sigma \;v}$$

where *N_t_* is the concentration of traps, σ the scattering cross section and *v* the carrier velocity.

**Figure 12:**
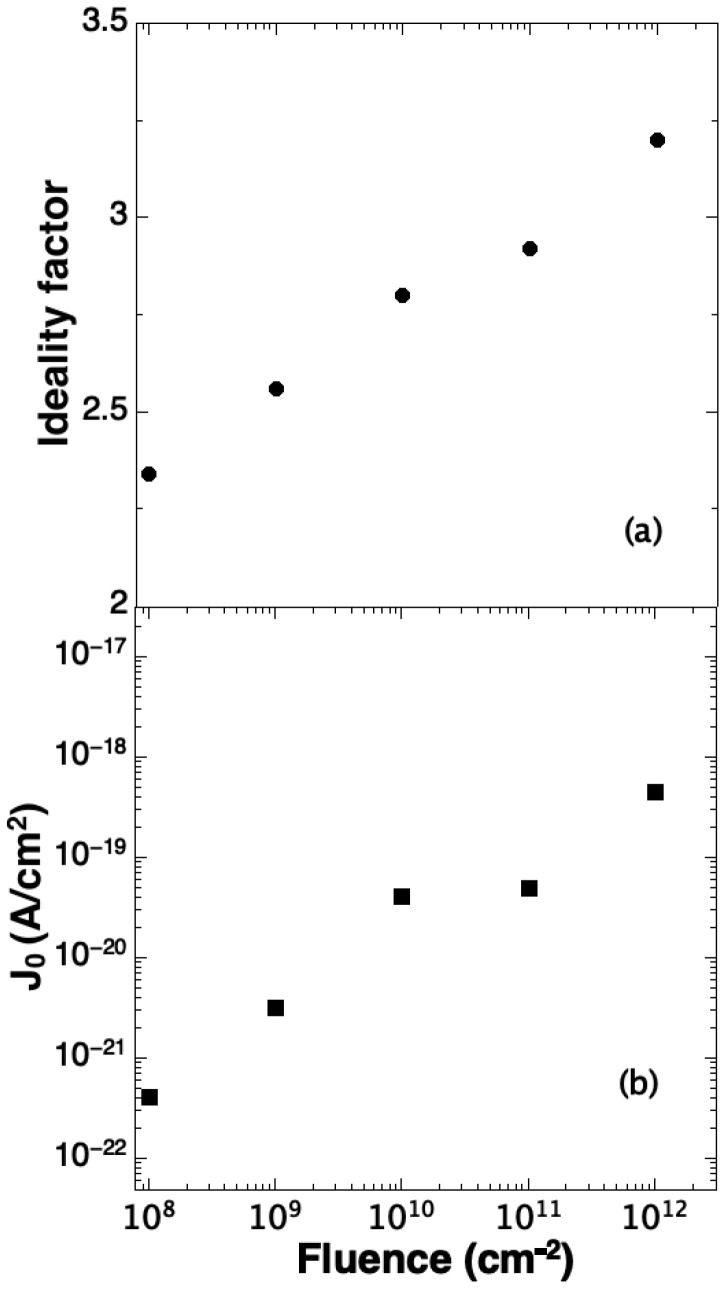
Ideality factor (**a**) and J_0_ parameter (**b**) versus ion fluence. In the upper scale, the total vacancy/cm^2^ is reported.

The obtained values of *n*, calculated by Equation (1) in the bias range where exponential trend holds, are reported in Figure 12a vs. the irradiation fluence. This parameter is about 2.3 in the unirradiated diode and increases with fluence reaching the value *n* = 3.2 in the highest fluence irradiated diode. Pezzimenti et al. [39] shows that the presence of defects considerably increases the ideality factor, and that, when the defect concentration becomes higher than 10^15^ cm^−3^, the value of the ideality factor increases and can reach values close to 3.0, typical of leaky diodes.

From the I–V curves, we have extracted the pre-exponential *J_0_* parameters. The obtained values are reported in Figure 12b vs. the irradiation fluence: it is 4 × 10^−22^ A/cm^2^ in the unirradiated diode and increases to 5 × 10^−19^ A/cm^2^ in the highest fluence irradiated device. From Equation (2), we deduce that the change in J_0_ can be associated to a change in the change of in *w* and *τ*_0_, as it is improbable any change of n_i_.

Moreover, as reported in the literature [38,41], the change of w, can be extracted from the barrier capacitance C_0_ (capacitance at V = 0) of the p-n junction. The maximum change of this parameter, as will be shown later, is of about a factor 3.5, then an increase of w of the same factor can be assumed. All these observations indicate that the measured increase of J_0_ of more than three orders of magnitude is related to the decrease of the carrier lifetime *τ*_0_ and then to the increase of defects concentration. This behavior has been confirmed by DLTS measurements, which will be presented in the following. Some authors of the present paper obtained similar results after irradiation of p-n junction with 600 Kev He ions [38].

At high voltage, the forward I–V characteristics (Figure 11b) tend to become flat as the effect of series resistance becomes dominant [39]. All the characteristics show a similar trend, with a decrease of current for the same bias value when the irradiation fluence increases. This effect is ascribed to the rise of diode series resistance (R_s_), and it was also observed in irradiated p-n junctions [21,22,38] and Schottky diodes [5,42] and was associated to the defects created by irradiation.

In fact, the created defects exhibit acceptor-like behavior [21,43], then compensation effects occur with a decrease of carrier concentration and an increase of resistivity.

The overall diode series resistance (R_s_) was extracted from the slope of the forward I–V curves in the high voltage region in the range 7.0 ÷ 8.0 V (not shown). The calculated values of R_s_ range from 1.5 Ohm for the unirradiated diodes to 5.8 Ohm for the highest fluence 10^12^ ions/cm^2^ irradiated device.

**Figure 13:**
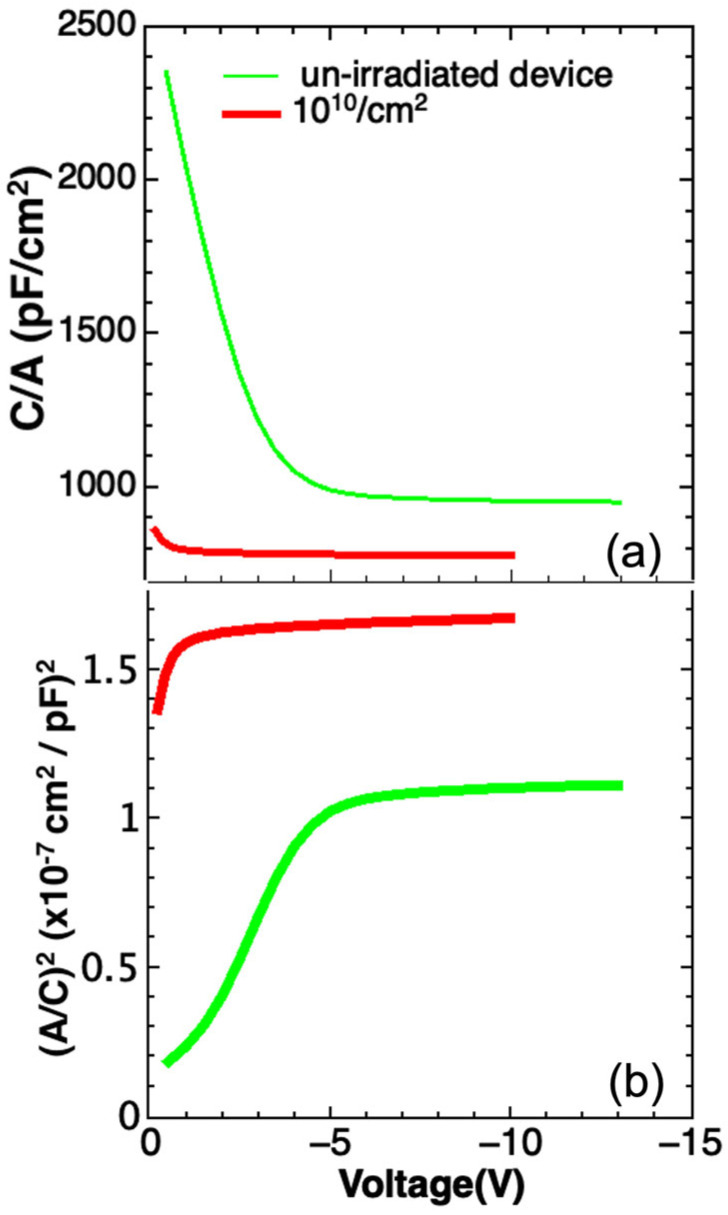
(**a**) C−V curves for unirradiated and irradiated detectors at a fluence of 10^10/^cm^2^ and (**b**) (A/C)^2^ vs. V plot for the same detectors.

To evidence the occurrence of change in the carrier concentration, C-V measurements were performed on unirradiated and on some irradiated devices. The C-V curves of unirradiated diode and of a device irradiated with 24.2 MeV ^27^Al ions at a fluence of 10^10^ Al/cm^2^ are reported in Figure 13.

Typical trends of C-V are observed in the unirradiated and irradiated detectors (Figure 13a) and the value of carrier concentration (N_D_-N_A_), extracted from the (A/C)^2^-V curves [31] (Figure 13b), is 9.5 × 10^13^ cm^−3^, very close to the nominal dopant concentration value- 8 × 10^13^ cm^−3^. After irradiation with 24.2 MeV ^27^Al ions at a fluence of 10^10^ Al/cm^2^ a change of C-V is observed (Figure 13a): The capacitance decreases, and a different trend was observed at a lower bias (2.0 V) with respect to unirradiated diode (4.0 V), indicating that for this bias all the n-type layer is already depleted. The carrier concentration calculated from the corresponding (A/C)^2^-V curve reported in Figure 13b is 3.1 × 10^13^ cm^−3^, a factor three lower than in the unirradiated sample.

C-V measurements performed in devices irradiated at high fluence show that the capacitance depends much less on the applied voltage, probably due to the complete depletion of the n^−^ region already at zero bias. Almost certainly at high fluence irradiation the carrier concentration could continue to decrease, and simultaneously, the mobility could be significantly reduced.

The changes of the pre-exponential parameter J_0_ and of the series resistance (or epilayer resistivity) are certainly both related to deep levels associated with defects generated in the material by irradiation. To investigate that, DLTS measurements were carried out in the device irradiated with 24.2 MeV ^27^Al at a fluence of 10^10^ ions/cm^2^.

**Figure 14:**
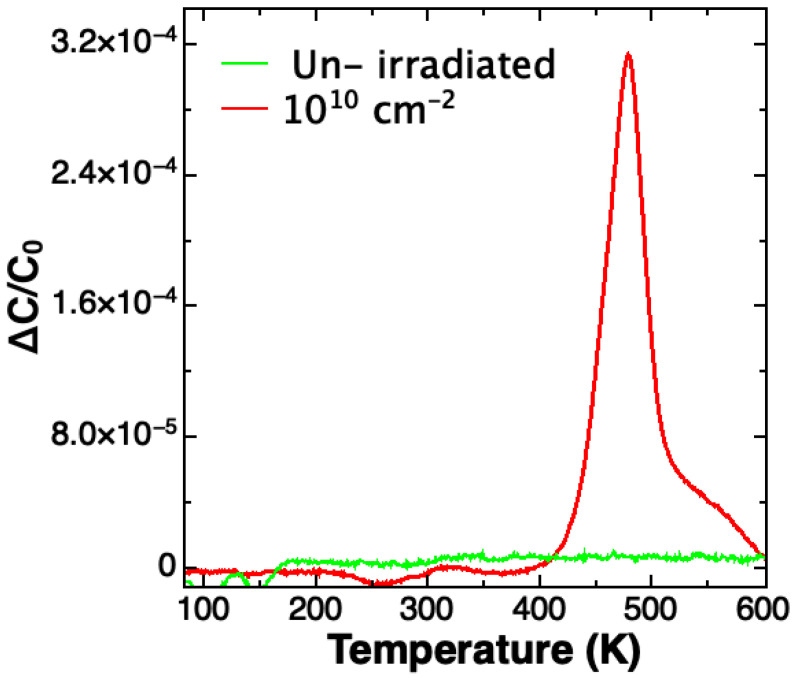
DLTS spectra of unirradiated and of 10^10^ cm^−2^ irradiated diodes.

SULA double boxcar spectrometer was used for DLTS measurements. The spectra were acquired in the temperature range 100–750 K by using rate windows in the range 2–200 s^−1^. In Figure 14 we report the obtained spectra measured in unirradiated and in 10^10^ Al/cm^2^ irradiated device.

The spectrum of unirradiated diode does not show any peaks, indicating the low concentration of processing defects. A main peak at 480 K with a broad band appears in the DLTS spectrum after irradiation at 1.0 × 10^10^ ions/cm^2^. The energy associated with the peak, determined by the Arrhenius plot obtained by performing measurements at different rate windows, is about 1.0 ± 0.05 eV below the conduction band. The concentration of this trap is about 10^10^ traps/cm^3^. Literature works relate these peaks (called RD_1/2_) to radiation damage: Their energies are in the range 0.9–1.0 eV [44,45].

Regarding the microstructure of the defect associated with this trap, the exact correlation with a specific structural defect has not been yet established, but a V_Si_ or an antisite could be responsible for these centers [43,45].

The DLTS spectra agree with the trend of the J_0_ parameter versus the irradiation fluence shown in Figure 11: The increase of J_0_ with fluence is to be imputed to the accumulation of point defects.

## 7. Summary

In this work we have reported an extended study on the radiation damage of SiC detectors.

In the first part, a comparison between the radiation damage of Si and SiC detectors has been performed. The detectors have been irradiated with ^16^O ions, stopped into the active area, and the same ions have been used for CCE evaluation (right after irradiation). While the CCE is similar between the two detectors, the resolution of the silicon detector deteriorates even at very low irradiation fluences (10^9^ ions/cm^2^). The silicon detector signal shows a very asymmetric peak. The silicon carbide detector instead shows a small increase of the resolution even at larger fluences (10^13^ ions/cm^2^), and the signal is always symmetric.

In the second part of the experiment, a heavier ion (^27^Al instead of ^16^O) was used to test the SiC detectors at higher radiation damage, and even in this case, the signal was symmetric at high irradiation fluences. The CCE has been evaluated many hours after irradiation by exploiting a ^148^Gd alpha source. Without collimation a second signal appears due to the edge structure of the detector and the CCE is always larger than 70% even for the higher fluence of 10^11^ ions/cm^2^. The CCE is also always increasing with the increase of the bias of the detector, and no saturation is observed until 700 V.

The same detectors irradiated with ^27^Al have been characterized by I–V, C-V and DLTS. It has been observed that the radiation damage produces an increase of the point defects observed by DLTS (RD_1/2_), and this defect induces both an increase of the leakage current of the detector, a shift of the forward characteristics and a reduction of the CCE of the detector. Furthermore, even an increase of the ideality factor and a decrease of the doping concentration of the epitaxial layer has been observed at high irradiation fluences.

Even if the electrical measurements show an increase of the leakage current due to the formation of point defects, the detector parameters (CCE and resolution) in SiC detectors are still good, showing a much higher radiation hardness with respect to silicon.

## Figures and Tables

**Table 1:** Energy threshold for the production of a PKA.

*Material*	Si	Diamond	GaN	4H SiC
***Property***	MCz, FZ, epi	Polycrystal	Single crystal	Epitaxial
***Displacement [eV]***	13–20	43	15	25

## Data Availability

The data are available on request.
